# Prevalence and long-term change in alcohol consumption: results from a population-based cohort in Southern India

**DOI:** 10.1186/s13033-024-00650-w

**Published:** 2024-10-10

**Authors:** S. Mahasampath Gowri, Antonisamy Belavendra, Senthil K. Vasan, S. Keerthi, Sven Andreasson

**Affiliations:** 1 Department of Biostatistics, Christian Medical College, Vellore, 632002 India; 2 MRC Life Course Epidemiology Centre, University of Southampton, Southampton, UK; 3 Oxford Centre for Diabetes, Endocrinology and Metabolism, University of Oxford, Oxford, UK; 4 Department of Global Public Health, Karolinska Institutet, Stockholm, Sweden

**Keywords:** Prevalence, Long-term change, Harmful and hazardous use, Help-seeking behavior

## Abstract

**Background:**

Alcohol consumption in India is below the global average, with limited data on long-term effects. The current study aims to examine changes over time among alcohol consumers, the pattern of drinking and help-seeking for alcohol problems among South Indian men.

**Method:**

Data on the intake of various alcohol types were collected through standard questionnaires in two adult follow-ups [Baseline: 1998–2002, Follow-up: 2016–2019] from male participants in the Vellore birth cohort (VBC). Alcohol intake was converted to weekly standard drink units for analysis. Data on drinking patterns using the Alcohol Use Disorder Identification Test (AUDIT) and information on help-seeking among problem drinkers were collected during follow-up. Socio-demographic associations with alcohol consumption were determined using logistic regression.

**Results:**

The prevalence of alcohol consumption was 54.5% and 47.7% at the baseline and follow-up, respectively. Over two decades, 12% of men reported to have newly started drinking and 18% quit drinking. Lower education and lower socio-economic status (SES) were the strongest predictors of alcohol consumption. The AUDIT assessment among drinkers reported hazardous drinking of 38.4%, harmful drinking of 4.7% and 3.7% probable alcohol dependence. Among the persons with high AUDIT scores, 25% were concerned about high consumption, and 9% sought help to stop their alcohol consumption.

**Conclusion:**

Our results showed a decline in alcohol consumption in this cohort over two decades. Among drinkers, a high proportion report hazardous and harmful consumption. Low levels of education and SES are significant predictors of alcohol consumption. A low proportion of help-seeking reflects alcohol-related stigma in the community.

**Supplementary Information:**

The online version contains supplementary material available at 10.1186/s13033-024-00650-w.

## Background

Alcohol is a psychoactive substance, which is the seventh leading risk factor for death and disability [1–5]. In younger age groups (20–38 years), 13.5% of deaths globally were attributable to alcohol consumption [6]. While some parts of Europe, notably the Mediterranean, show a decline in alcohol consumption, lower-middle-income countries experience a 38% increase over 15 years [7]. In India, the National Family Health Survey (NFHS-4) reported alcohol use in 29% of men and 1% of women aged 15–49 years, respectively [8]. Recent studies from Tamil Nadu report a prevalence of alcohol consumption in the range of 16.8–42.7% [9–12].

The alcohol policies in India are state-specific, and consumption reflects the diversity and culture within the nation. Some Indian states, namely Tamilnadu, Andhra Pradesh, Haryana, Kerala, Lakshadweep and Manipur, have had strict alcohol prohibition policies, subsequently lifted. In contrast, the states of Bihar, Gujarat, Mizoram and Nagaland are still under prohibition policies. In Tamil Nadu and Kerala, the alcohol trade is controlled by state alcohol monopolies. The Tamil Nadu alcohol monopoly (TASMAC) revenue was reported at around 36.4 billion Indian rupees in 2003–2004 and increased to 440 billion rupees in the financial year 2022–2023 [13, 14]. Likely reasons contributing to this 12.1-fold increase would be increased affordability and easy access to alcohol [15].

The increased access to alcohol has contributed to high rates of hazardous and harmful drinking in India, especially among males. Existing nationwide surveys and scattered reports from India estimate higher alcohol prevalence in men aged 35–60 years, where lower SES, education levels, and unemployment are essential factors for alcohol consumption [16–18]. Despite the reported higher prevalence, limited information exists on help-seeking behaviour [9, 17, 18].

The current study aims to examine the (i) prevalence, (ii) long-term change in alcohol consumption and (iii) hazardous drinking and help-seeking behaviour in a representative population sample from South India.

## Methods

**Fig. 1:**
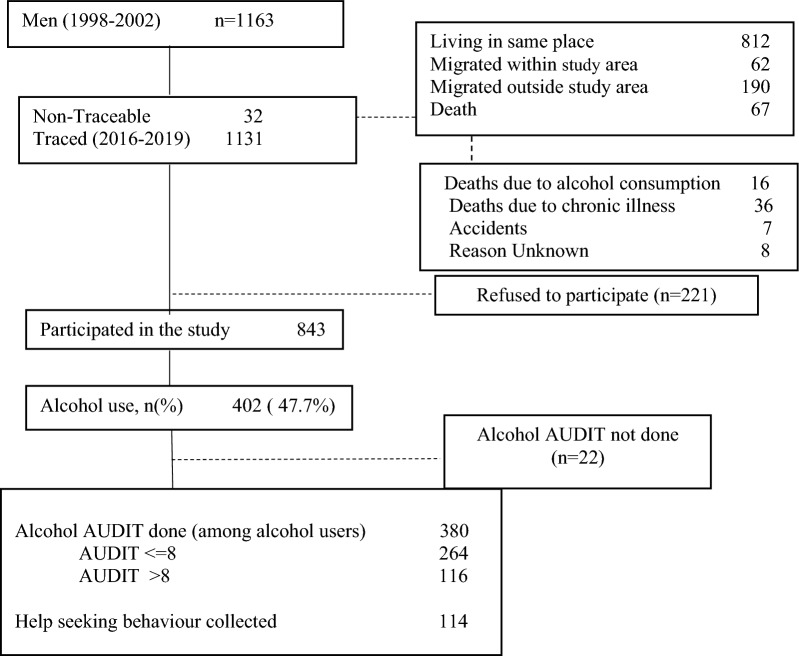
Flowchart presenting the follow-up of participants from 1998–2002 to 2016–2019

We used data from two adult follow-ups of the Vellore birth cohort (VBC). The cohort includes individuals born within representative areas of Vellore town and adjoining rural villages in Tamilnadu, India, from 1969 to 1973. The cohort was followed during different stages of life course, including birth, infancy (3 months), childhood (6.5 years), adolescence (15 years) and three stages of adulthood (26 years, 43 years and 45 years). The growth measurements during all the phases, along with lifestyle and non-communicable diseases (NCD) risk factors in adulthood, were collected using standardized instruments, and investigations using trained personnel. A detailed cohort description is provided elsewhere [19, 20]. The current analysis used data from 2218 cohort members (men = 1163, women = 1055) surveyed as young adults during 1998–2002 (baseline) and 1601 (men = 843, women = 758) who were subsequently followed during 2016–2019. Alcohol consumption was not reported among women during both phases of follow-up, and therefore, the current study is limited to men only. The flowchart presents the tracing and follow-up details among the men enrolled and followed in the study (Figure 1). Trained health workers with long-time involvement in the community collected the information on alcohol consumption, ensuring the data's accuracy. The health workers received training in verbal autopsy to determine the cause of death from baseline to follow-up. Out of the 67 deaths reported during follow-up, 16 (23.9%) were attributed to illnesses, accidents, or suicides resulting from alcohol consumption.

Standard questionnaires of the same were administered to obtain information on marital status, education, occupation, socioeconomic status (SES), place of residence, alcohol consumption, tobacco use and smoking at both times. Marital status was defined as unmarried, married, divorced, or widowed. Educational status spanned four groups, from no schooling to a professional qualification. Occupation was classified into seven groups, ranging from unemployed to professional. Socio-economic status (SES) was determined by the household's material possessions (such as a mattress, pressure cooker, chair, cot/bed, table, clock/watch, electric fan, bicycle, radio, television, moped/scooter/motorcycle, car/jeep, water pump, bullock cart, thresher, tractor, refrigerator, telephone, sewing machine, mobile phone, computer, internet, air cooler, air-conditioner). A composite score using household items was derived through principal component analysis (PCA). The resulting component score was categorised into quartiles. During the follow-up, physical activity was collected using IPAQ guidelines [21] for the baseline and GPAQ guidelines [22]. They were later categorised in three groups from low to vigorous activity for analysis. Smoking was classified as smokers and non-smokers based on the use of cigarettes, beedis (thin roll of wrapped tobacco) and cigars. Chewing tobacco leaves was classified as non-smoking tobacco use. Only 1% of the use of pan/zarda and ganja was reported in both phases, and these users were eliminated from the analysis.

Data on weekly alcohol consumption level included frequency and volume intake of spirits, beer and wine. These were converted into standard drink units (SDU) of alcohol per week (1 unit = 12 g; this corresponds to 40 ml of spirits (40% alcohol), 300 ml of beer (5% alcohol), or 120 ml of wine (12% alcohol). Alcohol consumption was categorised as none (0 units), low (≤ 7 units/week), moderate (8–21 units/week) and heavy (≥ 21 units/week) and dichotomised into consumers and non-consumers of alcohol. The analysis of change in alcohol consumption was approached in two ways: (i) computing the difference in alcohol consumption levels in standard units from baseline to follow-up by subtracting the values, and (ii) categorising alcohol use at baseline and follow-up into four groups: never (no alcohol consumption in both periods), started (non-consumer in the baseline but started consuming in the subsequent follow-up), continued (consumer in both periods), and stopped (ceased alcohol consumption in follow-up).

Harmful or hazardous alcohol use was assessed using the Alcohol Use Disorder Identification Test (AUDIT), which has been validated for use in South India [23, 24]. AUDIT was administered later during the study period; thus, the data were collected for 380 (94.5%) alcohol consumers. The scores were categorised as low-risk drinking (Zone I: score 0–7), hazardous drinking (Zone II: score 8–15), harmful drinking (Zone 3: score 16–19) and probable alcohol dependence (Zone IV: score 20 and above). Data on the concerns regarding alcohol use and help-seeking behaviour were collected through a validated questionnaire from 114 participants with an AUDIT score exceeding 8.

The presence of chronic disease was defined as whether the participants had any of the conditions, such as type-II diabetes or, hypertension or hypertriglyceridemia. Type-2 diabetes was defined as fasting glucose concentration (≥ 7.0 mmol/l) or glucose concentration 120 min (≥ 11.1 mmol/l) at the time of the survey or on treatment for type-2 diabetes [25]. Hypertension was defined as systolic blood pressure (≥ 140 mm Hg) or diastolic blood pressure (≥ 90 mm Hg) or currently on treatment for hypertension [26]. Hypertriglyceridemia defined as plasma triglyceride concentration (≥ 1.7 mmol/l) [27].

### Statistical analysis

Categorical data were expressed as frequencies and percentages, while continuous variables were presented as mean and standard deviation (SD) or median (interquartile range) as appropriate. Alcohol prevalence was compared using the z-test for proportions, and the absolute change in alcohol distribution levels was assessed using the sign rank test. Binary logistic regression was used to determine the association between alcohol use and demographic factors. Fisher-Yates transformation normalised actual consumption levels, which were then compared among demographic variable categories using t-tests and ANOVA. Absolute changes in consumption levels were skewed and compared among demographic variable categories using the Mann–Whitney U and Kruskal–Wallis tests. Ordinal logistic regression was used to determine the association of categorised trends in alcohol consumption and demographic factors. Other categorical associations were assessed using the chi-square test. A p < 0.05 was considered significant. All analyses were conducted using STATA/IC 16.0 (StataCorp, College Station, Texas 77845, USA).

## Results

### Prevalence and long-term change in alcohol consumption

In the present follow-up, 72.4% of the study participants from the baseline were included. There were no differences in alcohol consumption levels among participants and non-participants at baseline. The mean (SD) age at baseline and follow-up were 28.1 (1.2) and 45.9 (1.1) years respectively. The alcohol consumption prevalence at baseline was 54.5% (95% CI 51.6, 57.4)] and significantly decreased to 47.7% (95% CI 44.3, 51.1) during the follow-up (Z = 3.01, p < 0.01). In general, 34.3% were non-consumers of alcohol through the two decades, and 35.7% of individuals continued to drink in both the phases. Among the non-consumers at baseline, 25.9% began consuming alcohol during follow-up, whereas 33.6% of the baseline consumers quit drinking during follow-up. The median (IQR) alcohol consumption per week at baseline (n = 643) and follow-up(n = 402) are 4.53 (2.37, 7.23) SDU and 4.73 (4.73, 9.47) SDU, respectively. The maximum consumption during baseline was 187.13, and the follow-up was 110.46 SDU per week.

The type of alcohol consumption changed over time. Branded spirits largely replaced locally made spirits and made up the bulk, over 90%, of all reported alcohol consumption at follow-up. Locally made spirits reduced from 6.6 to 0.4%. Consumption of beer was reported by 37.5% at baseline and 3.2% at follow-up. Wine consumption was rare; 2.1% at baseline, dropping to 0.5% at follow-up.

### Factors associated with long-term change in alcohol use

The long-term change among alcohol users was calculated as the absolute difference in alcohol consumption between two-time points (difference = follow-up—baseline). A positive difference signifies increased consumption at follow-up, while a negative difference indicates the opposite. The calculations do not include participants who were non-drinkers at both time points ( n = 289). The median(IQR) difference in alcohol consumption is 0.21(−2.49, 4.73) SDU per week with a minimum difference of -142.55 and a maximum difference of 84.42 for the alcohol consumers (n = 554). This difference in alcohol consumption was statistically significant (Z = −2.30,p = 0.021). An increased consumption was reported for participants who were unmarried, with lower education and lower SES, rural residents, smoking, non-smoking tobacco use and low levels of physical activity at the follow-up time point. These differences in level of consumption were statistically significant for marital status, place of residence, smoking and non-smoking tobacco use (Table 1).

**Table 1:** Change in level of alcohol (SDU per week) according to socio-demographic variables (n = 554)

Socio-demographic variables	n	Median	IQR	*P*
Marital status^a^				
Unmarried	11	4.73	2.57, 17.09	0.043
Married	529	0.21	−2.49, 4.73	
Widowed/divorced	14	−1.46	−2.49, 17.2	
Education^a^				
No schooling	26	2.10	−3.42, 9.47	0.308
Middle school completion	222	1.22	−2.49, 6.80	
Higher secondary	256	0.00	−2.49, 4.61	
Graduates	50	−0.22	−2.37, 3.65	
Occupation^a^				
Unemployed & unskilled manual labour	139	0.21	−2.89, 5.02	0.100
Semi-skilled manual labour	250	2.17	−2.49, 4.73	
Skilled manual labour	100	−0.14	−2.49, 2.80	
Trained/clerical & professional	65	−0.98	−2.49, 2.37	
Socio-economic status (quartiles)^a,b^				
1 (lowest)	150	2.24	−2.49, 6.97	0.154
2	140	1.07	−2.49, 4.73	
3	141	−0.67	−2.49, 3.74	
4 (Highest)	123	−0.13	−2.49, 3.94	
Place of residence^c^				
Rural	308	2.09	−2.49, 4.73	0.002
Urban	246	−0.59	−2.49, 3.65	
Smoking^c^				
Yes	176	2.24	−2.37, 8.13	0.004
No	378	−0.13	−2.49, 4.61	
Non-smoking tobacco^c^				
Yes	14	5.00	2.24, 25.51	0.005
No	537	0.11	−2.49, 4.73	
Physical activity^a,d^				
Low	59	0.21	−2.49, 3.57	0.836
Moderate	163	0.00	−2.49, 4.73	
High	332	0.27	−2.49, 4.73	

IQR = (25th percentile,75th percentile); Change is the difference in alcohol consumption calculated as (Follow-up – Baseline alcohol) SDU per week for alcohol consumers at any one time point

*P* values based on ^a^ Kruskal Wallis test was used

^b^Socio -economic status is reported in quartiles using principal component analysis [PCA] of all the material possession

^c^rank sum test was used

^d^GPAQ during follow-up [2016–2019]

A significant proportion (35.7%) of cohort participants continued to drink throughout both phases. A majority of the unmarried men(56%) were non-consumers in both phases, whereas the continuation of alcohol consumption was more common among married participants (36%). The non-consumption or discontinuation of alcohol at follow-up was reported higher for higher education (72.1%), skilled manual labourers (63.5%)/professional (70.6%) occupation and SES ≥ third quartile (58.0%). Discontinuation of alcohol consumption was associated with urban residents and non-smokers. Contrastingly, lower education (59.5%), unemployment (56.1%), and SES ≤ 2nd quartile (54.3%) were associated with continuing alcohol consumption throughout 18 years of follow-up or initiating alcohol consumption during the follow-up phase. Physical activity was not associated with a change in alcohol practice (Additional Table 1).

### Factors associated with alcohol use

The association between between demographic factors and alcohol consumption was also studied. Alcohol consumption was associated with lower levels of education and socioeconomic status, with smoking and with low levels of physical activity. The associations remained the same for both time points (Table 2). The risk for alcohol consumption was significantly lower in semi-skilled labour compared to professionals/skilled labours at baseline [AOR(95% CI) 0.43 (0.23, 0.78)], whereas during follow-up semi-skilled labour [AOR (95% CI) 1.91 (1.01, 3.30)] had a higher risk compared to professionals/skilled labours. Similarly, the rural residents had lower risk for alcohol consumption at baseline [AOR (95% CI) 0.76 (0.56, 1.02)] with increased risk during follow-up [AOR (95% CI) 1.45 (1.06, 2.01)] compared to urban residents.

**Table 2:** Association of alcohol use (yes/no) and socio-demographic variables

Socio-demographic variables	Baseline [1998–2002]				Follow-up (2016–2019)			
	Total subjects	Alcohol users (n = 634)			Total subjects	Alcohol users (n = 402)		
	(n = 1163)	n	%	AOR [95% CI]^a^	(n = 843)	n	%	AOR [95% CI]^*a*^
Marital status								
Married	597	351	55.36	1.28 [0.97, 1.68]	802	384	95.52	1.42 [0.58, 3.52]
Widowed/divorced^b^	6	6	0.95	NA	16	9	2.24	NA
Unmarried	560	277	43.69	1.00	25	9	2.24	1.00
Education								
No schooling	55	29	4.57	1.14 [0.52, 2.52]	42	25	6.22	1.34 [0.53, 3.35]
Middle school completion	405	240	37.85	1.43 [0.87, 2.36]	312	176	43.78	1.66 [0.91, 3.02]
Higher secondary	549	300	47.32	1.47 [0.93 2.31]	385	172	42.79	1.38 [0.78, 2.37]
Graduates	154	65	10.25	1.00	104	29	7.21	1.00
Occupation								
Unemployed & unskilled manual labour	36	15	2.37	0.83 [0.35, 1.99]	205	115	28.61	1.60 [0.86, 3.00]
Semi-skilled manual labour	305	146	23.03	0.43 [0.23, 0.78]^**^	345	189	47.01	1.91 [1.11, 3.30]^*^
Skilled manual labour	719	421	66.4	0.87 [0.52, 1.47]	167	61	15.17	1.04 [0.58, 1.85]
Trained/clerical & professional	103	52	8.2	1.00	126	37	9.2	1.00
Socio-economic status (quartiles)^c^								
1 [Lowest]	283	157	24.76	1.20 [0.76, 1.91]	211	131	32.59	1.44 [0.86, 2.39]
2	288	149	23.5	1.15 [0.75, 1.76]	211	106	26.37	1.06 [0.67, 1.69]
3	293	168	26.5	1.15 [0.77, 1.69]	217	90	22.39	0.86 [0.56, 1.34]
4 [Highest]	299	160	25.24	1.00	204	75	18.66	1.00
Place of residence								
Rural	617	306	48.26	0.76 [0.56, 1.12]	471	245	60.95	1.45 [1.06, 2.01]^*^
Urban	546	328	51.74	1.00	372	157	39.05	1.00
Smoking								
Yes	504	397	62.62	7.01 [5.26, 9.33]^***^	212	153	38.06	3.72 [2.58, 5.37]^***^
No	659	237	37.38	1.00	631	249	61.94	1.00
Non-smoking tobacco								
Yes	51	41	6.47	5.46 [2.56, 11.65]^***^	16	14	3.48	8.48 [1.83, 39.20]^**^
No	1107	589	92.9	1.00	824	386	96.02	1.00
Physical activity^d^								
Low	389	202	31.86	1.00 [0.70 1.45]	88	45	11.19	1.39 [0.83, 2.36]
Moderate	397	225	35.49	1.18 [0.85 1.64]	242	110	27.36	1.25 [0.87, 1.78]
High	387	207	32.65	1.00	513	247	61.44	1.00

^***^p<0.001; ^**^p<0.01; ^*^p<0.05

with-in column % were presented; AOR- Adjusted Odds Ratio

^a^AOR[95%CI] is presented using Binary Logistic regression

^b^category excluded from analysis due to low cell count

^c^Socio-economic status is reported in quintiles using principal component analysis [PCA] of all the material possession

^d^Physical activity collected using IPAQ during baseline [1998–2002] and GPAQ during follow-up [2016–2019]

Factors related to an increase in the level of alcohol consumption were assessed by comparing the distribution of alcohol levels among the demographical variables. Alcohol levels were higher among those with low education, unemployed or unskilled labour, lower SES, and smoking and non-smoking tobacco use compared to their counterparts. The results were similar in baseline and follow-up (Table 3).

**Table 3:** Levels of alcohol consumption (SDU) per week compared among demographic variables

Socio-demographic variables	n = 634	Baseline 1998–2002				*P*	n = 402	Follow-up 2014–2019				*P*
		Mean	SD	Median	IQR			Mean	SD	Median	IQR	
Marital status^a^												
Unmarried	277	6.80	13.34	2.67	2.16, 6.90	0.007	9	14.46	11.85	9.47	4.73, 18.41	NA
Married	351	8.82	17.95	4.73	2.37, 7.23		384	10.74	14.16	4.73	4.73, 9.47	
Widowed/divorced^b^	6	12.25	16.00	7.06	4.73, 9.72		9	27.06	34.15	9.47	4.73, 33.14	
Education^a^												
No schooling	29	12.49	14.08	7.23	2.63, 17.21	< 0.001	25	13.31	18.81	4.73	4.73, 13.68	0.001
Middle school completion	240	10.1	18.00	4.73	2.37, 9.13		176	13.81	18.02	4.73	4.73, 14.20	
Higher secondary	300	5.40	7.12	2.63	2.35, 6.90		172	8.84	10.61	4.73	4.40, 9.47	
Graduates	65	9.95	31.11	2.99	2.16, 5.13		29	7.36	9.34	4.73	2.37, 4.73	
Occupation^a^												
Unemployed & unskilled manual labour	15	16.78	44.61	2.49	2.49, 6.90	0.012	115	11.52	15.53	4.73	4.73, 14.20	0.027
Semi-skilled manual labour	146	10.06	18.06	4.86	2.37, 7.75		189	11.83	15.38	4.73	4.73, 9.47	
Skilled manual labour	421	7.33	14.26	3.94	2.37, 7.23		61	11.28	15.54	4.73	4.73, 9.47	
Trained/clerical & Professional	52	4.74	4.21	3.25	2.16, 6.74		37	6.72	8.23	4.73	2.37, 4.73	
Socio-economic status (quartiles)^c^												
1 Lowest	157	9.71	17.74	4.73	2.37, 8.74	0.099	131	12.63	17.58	4.73	4.73, 13.68	0.217
2	149	8.37	17.99	4.53	2.37, 7.23		106	11.33	14.49	4.73	4.73, 9.47	
3	168	7.34	13.62	4.53	2.16, 7.23		90	9.08	9.99	4.73	2.63, 9.47	
4 Highest	160	6.55	14.86	3.64	2.16, 6.90		75	11	15.57	4.73	4.60, 9.47	
Place of residence^d^												
Rural	306	7.03	10.9	4.53	2.37, 7.23	0.717	245	11.45	15.49	4.73	4.73, 9.94	0.808
Urban	328	8.84	19.73	4.53	2.26, 7.23		157	10.78	14.12	4.73	4.73, 9.47	
Smoking^d^												
Yes	397	10.1	19.41	4.86	2.49, 8.74	< 0.001	153	14.16	18.14	5.26	4.73, 15.78	< 0.001
No	237	4.51	6.61	2.49	2.16, 4.73		249	9.36	12.30	4.73	2.49, 9.47	
Non-smoking tobacco^d^												
Yes	41	5.07	4.07	4.73	2.37, 6.90	0.647	14	14.53	12.87	9.47	4.73, 33.14	0.181
No	589	8.16	16.63	4.53	2.37, 7.23		386	11.03	15.02	4.73	4.73, 9.47	
Physical activity^e^												
Low	202	6.70	7.75	4.53	2.37, 4.53	0.715	45	15.34	23.02	4.73	4.73, 15.78	0.637
Moderate	225	8.27	17.83	3.94	2.16, 3.94		110	11.76	16.54	4.73	4.73, 9.47	
High	207	8.89	19.73	4.73	2.37, 4.73		247	10.18	12.02	4.73	4.73, 9.47	

IQR = (25th percentile,75th percentile); The alcohol levels are z transformed by fisher’s Yates transformation to achieve normality; *NA* Not applicable

^a^P calculated based on ANOVA was used

^b^category excluded from analysis due to low cell count

^c^Socio -economic status is reported in quartiles using principal component analysis [PCA] of all the material possession

^d^P calculated based on Independent t test was used; ^e^ Physical activity collected using IPAQ during baseline [1998–2002] and GPAQ during follow-up [2016–2019]

A significant decreasing trend in alcohol consumption levels was observed with higher education, skilled/professional occupation and higher SES. A significantly increased risk in alcohol levels was reported for smoking [Baseline: AOR (95% CI) 6.34 (4.91, 8.18); Follow-up: AOR (95% CI) 3.58 (2.04, 4.93)], and for non-smoking tobacco use [Baseline: AOR (95% CI) 2.79 (1.65, 4.72); follow-up: AOR (95% CI) 5.25 (2.04, 12.48)]. Contrastingly, rural residents had low levels of drinking at baseline [AOR (95% CI) 0.71 (0.54, 0.93)] where an increase was reported during follow-up [AOR (95% CI) 1.42 (1.06, 1.92)]. No significant trends were observed in marital status or physical activity (Additional Tables 2 & 3).

### Alcohol use and chronic disease

Alcohol consumption was significantly more prevalent in groups with chronic disease (χ2 = 4.68,df = 1, p = 0.031). An increased level of alcohol consumption during the follow-up period was reported among the participants with chronic disease compared to the participants without chronic diseases (χ2 = 9.52, df = 3, p = 0.023). Furthermore, newly developed chronic disease during the follow-up period was more prevalent among the alcohol consumers compared to the non-consumers (46.02% vs 36.51%).

Over the decades, alcohol consumption was high in participants with chronic disease, whereas alcohol non-consumption or cessation was higher in participants without any chronic illnesses. Although a slight increase was observed in hazardous drinking and alcohol dependence among individuals with chronic disease, it was not statistically significant (Additional Table 4).

### Drinking behaviour among the alcohol users

The AUDIT analysis was done for 380 men who were alcohol consumers at follow-up. The median AUDIT score was 6.0 (3.0, 10.0), with slightly higher values in rural areas [7.0 (4.0, 11.0)] compared to urban areas [6.0 (3.0, 10.0)]. The scores suggest hazardous drinking (Zone II) in 32.9%, harmful drinking (Zone III) in 4.7% and probable alcohol dependence (Zone IV) in 3.7% of the cohort participants.

### Help-seeking behaviour

Problem awareness and help-seeking behaviour among the participants with AUDIT scores > 8 were studied (n = 114). The results showed that 25.4% were concerned about their high alcohol consumption, 12.3% considered seeking help, and 8.8% had sought help. A significant proportion of the individuals discussed the issue of high alcohol consumption with wife or family members (46.5%), while 13.2% did not discuss it with anyone. The participants with high SES were, to a more significant extent, aware of their consumption and were concerned. Among the heavy drinkers, there was a higher degree of concern about their drinking as well as a higher degree of discussing their drinking with family and friends and also of seeking help. Educational and occupational statuses were not associated with any help-seeking behaviour (Table 4).

**Table 4:** Association of help seeking behaviour among socio demographic variables and drinking patterns (n = 114)

Socio demographic variables and drinking patterns	How do you view your drinking?						Have you considered seeking help or have you sought help?						Have you discussed your drinking with anyone?							
	Have not thought about it n = 39		Aware that consumption is high, but not concerned n = 46		Aware that consumption is high, and concerned n = 29		Have not considered seeking help n = 90		Have considered seeking help, but have not done son = 14		Have sought help n = 10		None n = 15		Wife/family n = 53		Friends n = 13		Family & friends n = 33	
	n	%	n	%	n	%	n	%	n	%	n	%	n	%	n	%	n	%	n	%
Audit score																				
Hazardous drinking(n = 82)	30	36.59	27	32.93	25	30.49	68	82.93	7	8.54	7	8.54	11	13.41	43	52.44	10	12.20	18	21.95
Harmful drinking (n = 18)	5	27.78	10	55.56	3	16.67	15	83.33	2	11.11	1	5.56	2	11.11	8	44.44	0	0.00	8	44.44
Alcohol dependence (n = 14)	4	28.57	9	64.29	1	7.14	7	50.00	5	35.71	2	14.29	2	14.29	2	14.29	3	21.43	7	50.00
*P*	0.098						0.047						0.058							
Socio economic status (quartiles)^*a*^																				
1 (lowest) (n = 45)	19	42.22	18	40.00	8	17.78	38	84.44	4	8.89	3	6.67	8	17.78	24	53.33	3	6.67	10	22.22
2 (n = 30)	6	20.00	15	50.00	9	30.00	22	73.33	4	13.33	4	13.33	0	0.00	15	50.00	5	16.70	10	33.33
3 (n = 18)	6	33.33	9	50.00	3	16.67	13	72.22	3	16.67	2	11.11	3	16.67	6	33.33	4	22.2	5	27.78
4 (highest) (n = 21)	8	38.10	4	19.05	9	42.86	17	80.95	3	14.29	1	4.76	4	19.05	8	38.1	1	4.76	8	38.10
*P*	0.105						0.852						0.176							
Education																				
No schooling (n = 8)	2	25.00	4	50.00	2	25.00	6	75.00	2	25.00	0	0.00	2	25.00	4	50.00	1	12.50	1	12.50
Middle school completion (n = 59)	19	32.20	28	47.46	12	20.34	46	77.97	7	11.86	6	10.17	7	11.86	28	47.46	5	8.47	19	32.20
Higher secondary (n = 42)	15	35.71	13	30.95	14	33.33	33	78.57	5	11.9	4	9.52	4	9.52	18	42.86	7	16.7	13	30.95
Graduates (n = 5)	3	60.00	1	20.00	1	20.00	5	100.00	0	0.00	0	0.00	2	40.00	3	60.00	0	0.00	0	0.00
*P*	0.504						0.772						0.442							
Alcohol consumption standard units																				
Mild (≤ 7 units) (n = 39)	17	43.59	10	25.64	12	30.77	35	89.74	2	5.13	2	5.13	6	15.38	23	58.97	5	12.8	5	12.82
Moderate (8–21 units) (n = 36)	13	36.11	11	30.56	12	33.33	29	80.56	3	8.33	4	11.11	4	11.11	17	47.22	3	8.33	12	33.33
Heavy (> 21 units) (n = 39)	9	23.08	25	64.1	5	12.82	26	66.67	9	23.08	4	10.26	5	12.82	13	33.33	5	12.80	16	41.03
*P*	0.006						0.091						0.164							
Place of residence																				
Rural (n = 69)	28	40.58	28	40.58	13	18.84	53	76.81	10	14.49	6	8.70	12	17.39	38	55.07	7	10.10	12	17.39
Urban (n = 45)	11	24.44	18	40.00	16	35.56	37	82.22	4	8.89	4	8.89	3	6.67	15	33.33	6	13.30	21	46.67
*P*	0.079						0.671						0.004							

with-in-row % are presented;* P* calculated using chi- square test; ^*a*^ Socio -economic status is reported in quartiles using principal component analysis [PCA] of all the material possession

## Discussion

Alcohol consumption is an important public health issue globally and is a large contributor to morbidity and mortality from chronic disease as well as injuries. 23.9% of all deaths in this cohort were alcohol caused, indicating that alcohol constitutes an increasing health burden in this community. The results of our study show (i) a slight decrease in the prevalence of alcohol consumption as the cohort ages during a follow-up period of 17–18 years, (ii) alcohol consumption was associated with lower SES, lower levels of education, and rural residence and (iii) harmful or dependent drinking was reported among 8.4% (32/380) of participants (iv) heavy alcohol consumption with increased rates of hazardous or dependent drinking was reported among rural areas compared to urban areas (v) One-fourth of the participants were aware and concerned about their alcohol consumption habits and a vast majority of participants discussed the high alcohol consumption with family or friends (86.8% i.e., 99/114).

Our results are consistent with other survey studies from India in different places that have reported a consumption prevalence of more than 50% [17, 18, 28]. Our finding of higher alcohol consumption in rural areas, compared to urban, contradicts the findings of Gururaj et al. [29] who suggest that the increased availability of alcohol in rural areas and urbanization are reasons for increased prevalence [15, 29]. Alcohol quitting during follow-up was reported among 33.5% of drinkers at baseline, whereas 25.9% began to consume alcohol in the follow-up period. This result is in line with various studies that report a decreased level of alcohol consumption after the middle age of 45 years [16, 30]. Drinking behaviour over the life course varies considerably in different cultures. Most common however is that drinking is highest in young adulthood (ref WHO, 2024). The results from this study suggest that this is the case in South India as well. Typically, drinking is reduced as individuals assume parental roles and occupational responsibilities.

Our study confirms an association of lower SES with high alcohol consumption which aligns with an urban study in India which reported the same [16]. In international reviews no clear association between level of alcohol consumption and SES have been found. In contrast, the negative consequences of high alcohol consumption are stronger for people with low SES [31]. Our study reported smoking, lower education levels and unemployment/ unskilled labour as strong predictors of alcohol consumption. Interestingly, throughout the two follow-up periods, the continuation of alcohol consumption in this population is higher among unemployed participants or those with unskilled occupation, lower SES and lower education, which strongly re-confirms that the occupation, education and SES plays a vital role in alcohol consumption in this population [16, 31, 32]. Higher use of smoking was reported among the participants who continued alcohol consumption, which indicates an association between alcohol consumption and smoking [32]. The study found that prolonged and high levels of alcohol use were strongly associated with chronic disease, which aligns with our results established earlier in this cohort [33–35], and with other global studies [2, 36–38].

The AUDIT scores among the alcohol users indicated that 32.8% of the drinkers had a hazardous consumption, 4.7% harmful drinking and 3.7% probable alcohol dependence, where the estimates are similar to an earlier study done in the urban slums of Vellore in south India, where hazardous drinking was reported for 31% and alcohol dependence for 4% [38].

As is the case with heavy drinkers around the world, a small proportion in this study sought help. Similar findings have been reported from community studies in rural parts of India [18, 39]. The fear of social stigma due to addiction and lack of treatment facilities are important reasons for not considering to seek help [18, 40]. Concern about the level of alcohol consumption was high among hazardous drinkers (30.5%) compared to dependent drinkers (7.1%), where the dependence disorder can lead to faulty analyses and irrational decision-making. Participants with higher socioeconomic status and higher education were more concerned about their drinking than participants with lower socioeconomic status and lower education levels, most likely a consequence of higher awareness of alcohol as a health risk.

### Strengths and limitations

This is the first study to report long-term changes in alcohol consumption in a population of South India using validated questionnaires. We successfully followed 72.4% of the male participants from baseline to follow-up. A sensitivity analysis assessed sample representativeness between participants and non-participants during follow-up. Although statistically significant differences in age, SES categories, and place of residence were noted between participants and non-participants from baseline, these differences were minimal (Additional Table 5).

Our study was limited by using the AUDIT questionnaire only during the follow-up phase. The AUDIT data collection was done for 380 of 402 alcohol consumers. We acknowledge that the proportion of alcohol consumption may be an underestimate as it is self-reported, and alcohol consumption is often inaccurate and prone to reporting/recall bias. We believe this bias will be minimal due to the long-term rapport of health workers with the cohort participants in the study areas. Women in the community did not report any alcohol use, which may be due to the self-reporting bias due to socio-cultural norms. However, it is well known that alcohol consumption among women is very low among Asian Indians [1].

Our findings may not be nationally representative as it is single-centre based, yet they add valuable information on alcohol consumption to the dearth of data that is currently available from India.

## Conclusion

In conclusion, our study finds a prevalence of alcohol consumption among men of around 50% in this cohort. A slight decline in alcohol consumption is found as individuals progress into middle age. Low socioeconomic status (SES) was the strongest predictor of alcohol consumption. Alcohol-related deaths account for nearly a quarter of mortality within the cohort. Among drinkers, the proportions reporting harmful use or alcohol dependence were 4.7% and 3.7%, respectively, which are rates found in many countries around the world [6]. The majority of participants with alcohol use disorders in this study did not seek treatment for their drinking problems. The same observation has been found in many studies, reporting on the large treatment gap in the addiction field [41]. This poses significant challenges from a public health perspective. New forms of treatment and support need to be developed that are perceived as relevant and acceptable to people with these disorders as well as economically viable in jurisdictions with limited resources [42]. The results of this study underline the significance of alcohol as a risk factor for morbidity and mortality, as well as the dearth of available treatment for alcohol use disorders. Future research on this cohort could include qualitative studies focusing on alcohol-related stigma and mental health issues experienced by alcohol consumers and their families.

## Supplementary Information

Supplementary material 1.

## Data Availability

No datasets were generated or analysed during the current study.

## Abbreviations

VBC: Vellore birth cohort
AUDIT: Alcohol use disorder identification test
SDU: Standard drink unit
SES: Socio-economic status
TASMAC: Tamilnadu State Marketing Corporation
NCD: Non-communicable disease
PCA: Prinicipal component analysis
IPAQ: International Physical Activity Questionnaire
GPAQ: Global Physical Activity Questionnaire
SD: Standard deviation
IQR: Interquartile ranges
AOR: Adjusted odds ratio
CI: Confidence interval
