# Supplementary material for: Prevalence and long-term change in alcohol consumption: results from a population-based cohort in Southern India
Source: Int J Ment Health Syst. 2024 Oct 10;18:30. doi: 10.1186/s13033-024-00650-w (PMC11465489; doi:10.1186/s13033-024-00650-w)
Supplement: Supplementary file 1 — Supplementary material 1. [file 13033_2024_650_MOESM1_ESM.docx]

**Additional table 1: Association of change in alcohol use and socio demographic and life style variables in 2016-2019**

| **Socio-demographic variables** | **Alcohol consumption in both the phases (1998-2002) & (2016-2019) n (%)** | | | | | | | | | ***P*** |
| --- | --- | --- | --- | --- | --- | --- | --- | --- | --- | --- |
|  | **Never** | | **Stopped** | | | **Continued** | | **Started** | |  |
|  | **n=289** | | **n=152** | | | **n=301** | | **n=101** | |  |
|  | **n** | **%** | **n** | **%** | **n** | | **%** | **n** | **%** |  |
| **Marital status** |  |  |  |  |  | |  |  |  |  |
| Married (n=802) | 273 | 34.04 | 145 | 18.08 | 289 | | 36.03 | 95 | 11.85 | .004 |
| Unmarried ((n=25) | 14 | 56.00 | 2 | 8.00 | 3 | | 12.00 | 6 | 24.00 |  |
| Widowed/Divorced (n=16)*^a^* | 2 | 12.50 | 5 | 31.25 | 9 | | 56.25 | 0 | 0.00 |  |
| **Education** |  |  |  |  |  | |  |  |  |  |
| No schooling (n=42) | 16 | 38.10 | 1 | 2.38 | 21 | | 50.00 | 4 | 9.52 | < .001 |
| Middle school completion (n=312) | 90 | 28.85 | 46 | 14.74 | 129 | | 41.35 | 47 | 15.06 |  |
| Higher secondary (n=385) | 129 | 33.51 | 84 | 21.82 | 131 | | 34.03 | 41 | 10.65 |  |
| Graduates (n=104) | 54 | 51.92 | 21 | 20.19 | 20 | | 19.23 | 9 | 8.65 |  |
| **Occupation** |  |  |  |  |  | |  |  |  |  |
| Unemployed & Unskilled manual labour (n=205) | 66 | 32.20 | 24 | 11.71 | 84 | | 40.98 | 31 | 15.12 | <.001 |
| Semi-skilled manual labour (n=345) | 95 | 27.54 | 61 | 17.68 | 142 | | 41.16 | 47 | 13.62 |  |
| Skilled manual labour (n=167) | 67 | 40.12 | 39 | 23.35 | 46 | | 27.54 | 15 | 8.98 |  |
| Trained/clerical & Professional (n=126) | 61 | 48.41 | 28 | 22.22 | 29 | | 23.02 | 8 | 6.35 |  |
| **Socio-economic status (quartiles)*^b^*** |  |  |  |  |  | |  |  |  |  |
| 1 (lowest) (n=211) | 61 | 28.91 | 19 | 9.00 | 92 | | 43.60 | 39 | 18.48 | <.001 |
| 2 (n=211) | 71 | 33.65 | 34 | 16.11 | 77 | | 36.49 | 29 | 13.74 |  |
| 3 (n=217) | 76 | 35.02 | 51 | 23.50 | 70 | | 32.26 | 20 | 9.22 |  |
| 4 (highest) (n=204) | 81 | 39.71 | 48 | 23.53 | 62 | | 30.39 | 13 | 6.37 |  |
| **Place of residence** |  |  |  |  |  | |  |  |  |  |
| Rural (n=471) | 163 | 34.61 | 63 | 13.38 | 170 | | 36.09 | 75 | 15.92 | <.001 |
| Urban (n=372) | 126 | 33.87 | 89 | 23.92 | 131 | | 35.22 | 26 | 6.99 |  |
| **Smoking** |  |  |  |  |  | |  |  |  |  |
| Yes (n=212) | 36 | 16.98 | 23 | 10.85 | 127 | | 59.91 | 26 | 12.26 | < .001 |
| No (n=631) | 253 | 40.10 | 129 | 20.44 | 174 | | 27.58 | 75 | 11.89 |  |
| **Non-Smoking Tobacco** |  |  |  |  |  | |  |  |  |  |
| No (n=824) | 287 | 34.83 | 151 | 18.33 | 287 | | 34.83 | 99 | 12.01 | NA |
| Yes (n=16) | 2 | 12.50 | 0 | 0.00 | 12 | | 75.00 | 2 | 12.50 |  |
| **Physical Activity*^c^*** |  |  |  |  |  | |  |  |  |  |
| Low (n=88) | 29 | 32.95 | 14 | 15.91 | 41 | | 46.59 | 4 | 4.55 | .044 |
| Moderate (n=242) | 79 | 32.64 | 53 | 21.90 | 85 | | 35.12 | 25 | 10.33 |  |
| High (n=513) | 181 | 35.28 | 85 | 16.57 | 175 | | 34.11 | 72 | 14.04 |  |

with-in-rows % is presented ; *P* calculated using chi- square test; *^a^* category excluded from analysis due to low cell count ; *^b^* Socio -economic status is reported in quartiles using principal component analysis (PCA) of all the material possession; *^c^* GPAQ questionnaire is used to collect physical activity

**Additional table 2: Association of levels of alcohol consumption with socio demographic and life style factors in (1998-2002)**

| **Socio-demographic variables** | **Alcohol consumption levels (1998-2002), n%** | | | | | | | | **AOR [95% CI]*^a^*** | |
| --- | --- | --- | --- | --- | --- | --- | --- | --- | --- | --- |
|  | **None** | | **≤7 units** | | **8-21 units** | | **>21 units** | |  |  |
|  | **n=529** | | **n=458** | | **n=138** | | **n=38** | |  |  |
|  | **n** | **%** | **n** | **%** | **n** | **%** | **n** | **%** |  |  |
| **Marital status** |  |  |  |  |  |  |  |  |  |  |
| Married (n=597) | 246 | 41.21 | 247 | 41.37 | 80 | 13.4 | 24 | 4.02 | 1.21 [0.95, 1.54] |  |
| Widowed/Divorced (n=6)*^b^* | 0 | 0.00 | 3 | 50.00 | 2 | 33.33 | 1 | 16.67 | NA |  |
| Unmarried (n=560) | 283 | 50.54 | 208 | 37.14 | 56 | 10.00 | 13 | 2.32 | 1.00 |  |
| **Education** |  |  |  |  |  |  |  |  |  |  |
| No schooling (n=55) | 26 | 47.27 | 14 | 25.45 | 11 | 20.00 | 4 | 7.27 | 1.59 [0.78, 3.22] |  |
| Middle school completion (n=405) | 165 | 40.74 | 159 | 39.26 | 60 | 14.81 | 21 | 5.19 | 1.59 [1.01, 2.50] |  |
| Higher secondary (n=549) | 249 | 45.36 | 234 | 42.62 | 56 | 10.2 | 10 | 1.82 | 1.34 [0.89, 2.03] |  |
| Graduates (n=154) | 89 | 57.79 | 51 | 33.12 | 11 | 7.14 | 3 | 1.95 | 1.00 |  |
| **Occupation** |  |  |  |  |  |  |  |  |  |  |
| Unemployed & Unskilled manual labour (n=36) | 21 | 58.33 | 12 | 33.33 | 1 | 2.78 | 2 | 5.56 | 0.91 [0.41, 2.05] |  |
| Semi-skilled manual labour (n=305) | 159 | 52.13 | 94 | 30.82 | 40 | 13.11 | 12 | 3.93 | 0.50 [0.30, 0.86] |  |
| Skilled manual labour (n=719) | 298 | 41.45 | 312 | 43.39 | 86 | 11.96 | 23 | 3.20 | 0.88 [0.55, 1.39] |  |
| Trained/clerical & Professional (n=103) | 51 | 49.51 | 40 | 38.83 | 11 | 10.68 | 1 | 0.97 | 1.00 |  |
| **Socio-economic status (quantiles)*^c^*** |  |  |  |  |  |  |  |  |  |  |
| 1 (lowest) (n=283) | 126 | 44.52 | 103 | 36.40 | 40 | 14.13 | 14 | 4.95 | 1.33 [0.88, 2.00] |  |
| 2 (n=288) | 139 | 48.26 | 109 | 37.85 | 30 | 10.42 | 10 | 3.47 | 1.13 [0.78, 1.65] |  |
| 3 (n=293) | 125 | 42.66 | 124 | 42.32 | 36 | 12.29 | 8 | 2.73 | 1.07 [0.76, 1.50] |  |
| 4 (highest) (n=299) | 139 | 46.49 | 122 | 40.80 | 32 | 10.70 | 6 | 2.01 | 1.00 |  |
| **Place of residence** |  |  |  |  |  |  |  |  |  |  |
| Rural (n=617) | 311 | 50.41 | 226 | 36.63 | 65 | 10.53 | 15 | 2.43 | 0.71 [0.54, 0.93] ^*^ |  |
| Urban (n=546) | 218 | 39.93 | 232 | 42.49 | 73 | 13.37 | 23 | 4.21 | 1.00 |  |
| **Smoking** |  |  |  |  |  |  |  |  |  |  |
| Yes (n=504) | 422 | 64.04 | 199 | 30.20 | 33 | 5.01 | 5 | 0.76 | 6.34 [4.91, 8.18] ^***^ |  |
| No (659) | 107 | 21.23 | 259 | 51.39 | 105 | 20.83 | 33 | 6.55 | 1.00 |  |
| **Non-smoking Tobacco** |  |  |  |  |  |  |  |  |  |  |
| Yes (n=51) | 10 | 19.61 | 32.00 | 62.75 | 9 | 17.65 | 0 | 0.00 | 2.79 [1.65, 4.72] *^***^* |  |
| No (n=1107) | 518 | 46.79 | 424 | 38.3 | 128 | 11.65 | 37 | 3.34 | 1.00 |  |
| **Physical Activity*^d^*** |  |  |  |  |  |  |  |  |  |  |
| Low (n=389) | 187 | 48.07 | 144 | 37.02 | 48 | 12.34 | 10 | 2.57 | 0.93 [0.69, 121] |  |
| Moderate (n=387) | 162 | 41.86 | 168 | 43.41 | 42 | 10.85 | 15 | 3.88 | 1.04 [0.78, 1.40] |  |
| High (n=387) | 180 | 46.51 | 146 | 37.73 | 48 | 12.40 | 13 | 3.66 | 1.00 |  |

with-in-rows % were presented; AOR- Adjusted Odds Ratio; *^a^* AOR [95%CI] is obtained using ordinal logistic regression ; ^b^ category excluded from analysis due to low cell count; *^c^* Socio -economic status is reported in quartiles using principal component analysis (PCA) of all the material possession; *^d^* IPAQ questionnaire is used to collect physical activity

**Additional table 3: Association between alcohol consumption levels and socio demographic and life style factors in (2016-2019)**

| **Socio-demographic variables** | **Alcohol consumption levels 2016-2019 , n%** | | | | | | | | **AOR [95% CI]*^a^*** | |
| --- | --- | --- | --- | --- | --- | --- | --- | --- | --- | --- |
|  | **None** | | **≤7 units** | | **8-21 units** | | **>21 units** | |  |  |
|  | **n=41** | | **n=251** | | **n=93** | | **n=58** | |  |  |
|  | **n** | **%** | **n** | **%** | **n** | **%** | **n** | **%** |  |  |
| **Marital status** |  |  |  |  |  |  |  |  |  |  |
| Married (n=802) | 418 | 52.12 | 243 | 30.30 | 88 | 10.97 | 53 | 6.61 | 1.09 [0.47, 2.52] |  |
| Widowed/Divorced (n=16)*^b^* | 7 | 43.75 | 4 | 25.00 | 2 | 12.50 | 3 | 18.75 | NA |  |
| Unmarried (n=25) | 16 | 64.00 | 4 | 16.00 | 3 | 12.00 | 2 | 8.00 | 1.00 |  |
| **Education** |  |  |  |  |  |  |  |  |  |  |
| No schooling (n=42) | 17 | 40.48 | 13 | 30.95 | 8 | 7.05 | 4 | 9.52 | 1.52 [0.67, 3.45] |  |
| Middle school completion (n=312) | 136 | 43.59 | 100 | 32.05 | 40 | 12.82 | 36 | 11.54 | 1.88 [1.07, 3.35]^*^ |  |
| Higher secondary (n=385) | 213 | 55.20 | 114 | 29.61 | 43 | 11.17 | 15 | 3.90 | 1.42 [0.84, 2.41] |  |
| Graduates (n=104) | 75 | 72.12 | 24 | 23.08 | 2 | 1.92 | 3 | 2.88 | 1.00 |  |
| **Occupation** |  |  |  |  |  |  |  |  |  |  |
| Unemployed & Unskilled manual labour (n=205) | 90 | 43.90 | 73 | 35.61 | 23 | 11.22 | 19 | 9.27 | 1.64 [0.91, 2.95] |  |
| Semi-skilled manual labour (n=345) | 156 | 45.22 | 107 | 31.01 | 55 | 15.94 | 27 | 7.83 | 2.09 [1.25, 3.53] *^*^* |  |
| Skilled manual labour (n=167) | 106 | 63.47 | 41 | 24.55 | 11 | 6.59 | 9 | 5.39 | 1.12 [0.64, 1.95] |  |
| Trained/clerical & Professional (126) | 89 | 70.63 | 30 | 23.81 | 4 | 3.17 | 3 | 2.38 | 1.00 |  |
| **Socio-economic status (quartiles)*^c^*** |  |  |  |  |  |  |  |  |  |  |
| 1 (lowest) (n=211) | 80 | 37.91 | 73 | 34.60 | 38 | 18.01 | 20 | 9.48 | 1.28 [0.81, 2.04] |  |
| 2 (n=211) | 105 | 49.76 | 68 | 32.23 | 20 | 9.48 | 18 | 8.53 | 1.00 [0.65, 1.55] |  |
| 3 (n=217) | 127 | 58.53 | 61 | 28.11 | 19 | 8.76 | 10 | 4.61 | 0.78 [0.51, 1.19] |  |
| 4 (highest) (n=204) | 129 | 63.24 | 49 | 24.02 | 16 | 7.84 | 10 | 4.90 | 1.00 |  |
| **Place of residence** |  |  |  |  |  |  |  |  |  |  |
| Rural (n=471) | 226 | 47.98 | 150 | 31.85 | 61 | 12.95 | 34 | 7.22 | 1.42 [1.06, 1.92] ^*^ |  |
| Urban (n=372) | 215 | 57.80 | 101 | 27.15 | 32 | 8.60 | 24 | 6.45 | 1.00 |  |
| **Smoking** |  |  |  |  |  |  |  |  |  |  |
| Yes (n=212) | 59 | 27.83 | 79 | 37.26 | 46 | 21.70 | 28 | 13.21 | 3.58 [2.04,4.93] ^**^ |  |
| No (n=631) | 382 | 60.54 | 172 | 27.26 | 47 | 7.45 | 30 | 4.75 | 1.00 |  |
| **Non-Smoking Tobacco** |  |  |  |  |  |  |  |  |  |  |
| Yes (n=824) | 2 | 12.50 | 6 | 37.50 | 4 | 25.00 | 4 | 25.00 | 5.25 [2.04, 13.48] ^**^ |  |
| No (n=16) | 438 | 53.16 | 244 | 29.61 | 89 | 10.80 | 53 | 6.43 | 1.00 |  |
| **Physical Activity*^d^*** |  |  |  |  |  |  |  |  |  |  |
| Low (n=88) | 43 | 48.86 | 28 | 31.82 | 9 | 10.23 | 8 | 9.09 | 1.38 [0.87, 2.20] |  |
| Moderate (n=242) | 132 | 54.55 | 71 | 29.34 | 23 | 9.50 | 16 | 6.61 | 1.25 [0.91, 1.73] |  |
| High (n=513) | 266 | 51.85 | 152 | 26.63 | 61 | 11.89 | 34 | 6.63 | 1.00 |  |

with-in-rows % were presented; AOR- Adjusted Odds Ratio; *^a^* AOR [95%CI] is obtained using ordinal logistic regression ; *^b^* category excluded from analysis due to low cell count; *^c^* Socio -economic status is reported in quartiles using principal component analysis (PCA) of all the material possession; *^d^* GPAQ questionnaire is used to collect physical activity

**Additional table 4: Association of chronic disease with alcohol consumption and drinking patterns**

| **Alcohol consumption and drinking patterns** | **Chronic Disease** | | | | ***P*** |
| --- | --- | --- | --- | --- | --- |
|  | **Yes** | | **No** | |  |
|  | **n=511** | | **n=332** | |  |
|  | **n** | **%** | **n** | **%** |  |
| **Alcohol consumption** |  |  |  |  |  |
| No, n=441 | 252 | 49.32 | 189 | 56.93 | .032 |
| Yes, n=401 | 259 | 50.68 | 143 | 43.07 |  |
| **Levels of Alcohol consumption during 2016-2019** |  |  |  |  |  |
| None, n==441 | 252 | 49.32 | 189 | 56.93 | .034 |
| Mild <=7 units, n=251 | 153 | 29.94 | 98 | 29.52 |  |
| Moderate 8-21 units, n=93 | 63 | 12.33 | 30 | 90.04 |  |
| Heavy >21 units, n=58 | 43 | 80.41 | 15 | 40.52 |  |
| **Change in alcohol consumption from baseline to follow-up** |  |  |  |  |  |
| Never, n= 289 | 155 | 30.33 | 134 | 40.36 | .023 |
| Started, n=152 | 97 | 18.98 | 55 | 16.57 |  |
| Continues, n=301 | 197 | 38.55 | 104 | 31.33 |  |
| Stopped, n=101 | 62 | 12.13 | 39 | 11.75 |  |
| **Alcohol AUDIT - *Risk level scoring*** |  |  |  |  |  |
| Low-risk drinking: Zone - I 0-7, n= 223 | 142 | 56.13 | 90 | 63.83 | .502 |
| Hazardous drinking: Zone - II 8-15, n=125 | 88 | 34.78 | 41 | 29.08 |  |
| Harmful Drinking: Zone - III 16-19, n=18 | 13 | 50.14 | 5 | 3.55 |  |
| Alcohol Dependence: Zone - IV 20 & above, n=14 | 10 | 3.95 | 5 | 3.55 |  |
| with-in column % were presented ;  *P* calculated using chi- square test | | | | |  |

**Additional table 5: Sensitivity analysis of individuals (in 1998-2002) participated and not participated in the follow-up (2016-2019)**

| **Socio-demographic variables** | **Participated** | | **Not Participated** | | ***P*** |
| --- | --- | --- | --- | --- | --- |
|  | **n=843** | | **n=320** | |  |
|  | **n** | **%** | **n** | **%** |  |
| **Marital status** |  |  |  |  |  |
| Married | 453 | 53.74 | 144 | 45.00 | .017 |
| Unmarried | 387 | 45.91 | 173 | 54.06 |  |
| Widowed/Divorced | 3 | 0.36 | 3 | 0.94 |  |
| **Socio-economic status(quartiles)*^a^*** |  |  |  |  |  |
| 1 (lowest) | 204 | 24.20 | 79 | 24.69 |  |
| 2 | 212 | 25.15 | 76 | 23.75 | .022 |
| 3 | 228 | 27.05 | 65 | 20.31 |  |
| 4 (Highest) | 199 | 23.61 | 100 | 31.25 |  |
| **Place of residence** |  |  |  |  |  |
| Rural | 479 | 56.58 | 140 | 43.75 | <.001 |
| Urban | 364 | 43.42 | 180 | 56.25 |  |
| **Smoking** | 351 | 41.64 | 153 | 47.81 | .148 |
| **Non-smoking tobacco** | 34 | 4.03 | 17 | 5.31 | .518 |
| **Physical Activity*^b^*** |  |  |  |  |  |
| Low | 284 | 33.69 | 105 | 32.81 |  |
| Moderate | 260 | 30.84 | 127 | 39.69 | .007 |
| High | 299 | 35.47 | 88 | 27.50 |  |

% were calculated with-in column; *P* calculated using chi- square test; *^a^* Socio -economic status is reported in quartiles using principal component analysis (PCA) of all the material possession; *^b^* IPAQ questionnaire is used to collect physical activity
